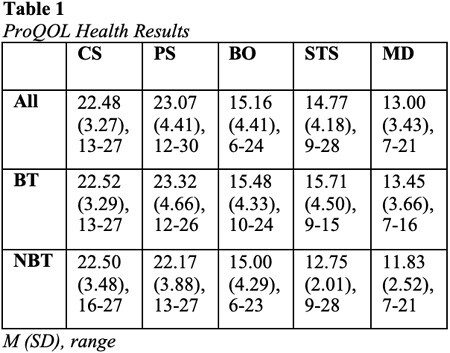# 98 Assessment of Burn Center Providers’ Professional Quality of Life

**DOI:** 10.1093/jbcr/irae036.097

**Published:** 2024-04-17

**Authors:** Christine M Grauer, Miranda L Yelvington

**Affiliations:** Arkansas Children's Hospital, Little Rock, Arkansas; Arkansas Children's Hospital, Little Rock, Arkansas

## Abstract

**Introduction:**

Professional Quality of Life encompasses concepts related to compassion satisfaction and compassion fatigue. Healthcare professionals experience compassion satisfaction as they enjoy caring for others and achieve success in their work. Compassion Fatigue has been associated with physical symptoms and illness among affected individuals, increased staff turnover, and decreased patient safety and satisfaction. The purpose of this study was to assess the professional quality of life of staff at one Burn Center.

**Methods:**

Multidisciplinary healthcare providers who care for adult and pediatric patients at a single Burn Center completed a demographic and Professional Quality of Life Health Measure (ProQOL Health) survey. The ProQOL Health consists of five subscales: Compassion Satisfaction (CS), Perceived Support (PS), Burnout (BO), Secondary Traumatic Stress (STS), and Moral Distress (MD). For each subscale, scores are defined as low ≤ 12, moderate 13-23, and high ≥ 24.

**Results:**

Forty-four participants completed this pilot survey of staff who care for people who sustained burn injuries. Twelve participants identified as non-burn team (NBT) members, and 32 identified as members of the burn team (BT). ProQOL Health subscale scores were calculated and analyzed. Mean, standard deviation, and range are reported for all participants and by subgroup (Table 1).

This cross-sectional study demonstrated moderate levels of CS, PS, BO, STS, and MD among healthcare providers who work directly with adult and pediatric burn survivors. An independent samples t-test was used to evaluate differences in subscale scores of the ProQOL Health based on burn team (n = 32) or non-burn team (n = 12) self-selection. There was a statistically significant difference in STS mean score between burn team and non-burn team members, t(40.63) = 2.81, p = .004. Mean BT STS scores (15.53 ± 4.54) were higher than mean NBT STS scores (12.75 ± 2.01). The BT mean STS score was 2.78 (95% CI, 0.78 to 4.78) higher than the NBT STS score. Groups were not significantly different for other subscales.

**Conclusions:**

Higher STS subscale scores among BT members may be due to repeated exposure to patients who have experienced traumatic and stressful events related to their burn injuries or the care they require.

**Applicability of Research to Practice:**

Interventions designed to address professional quality of life should be considered for all healthcare providers who care for burn survivors and not exclusively those who consider themselves members of the BT.